# Proactive personality, innovative work behavior, and employee performance: evidence from the insurance sector in Northern Cyprus

**DOI:** 10.3389/fpsyg.2026.1796666

**Published:** 2026-04-15

**Authors:** Ahmet Çukurovali, Ayşem Iyikal Çelebi, Serdal Işiktaş, Ahmet Melih Karavelioğlu

**Affiliations:** 1 Department of Business Administration, Faculty of Social Sciences and Humanities, Cyprus Health and Social Science University, Northern Cyprus, Güzelyurt, Türkiye; 2 Department of Business Administration, Faculty of Social Sciences and Humanities, Bahçeşehir Cyprus University, Northern Cyprus, Lefkoşa, Türkiye

**Keywords:** contextual performance, innovative work behavior, insurance sector, proactive personality, task performance

## Abstract

**Introduction:**

Proactive personality is increasingly recognized as a key individual difference that may drive innovative work behavior and enhance employee performance in dynamic service sectors. However, empirical evidence examining the interrelationships among proactive personality, innovative work behavior, task performance, and contextual performance remains limited, particularly in the insurance industry of small economies such as Northern Cyprus. This study aims to investigate these relationships among insurance employees.

**Methods:**

A cross-sectional survey design was employed. Data were collected in 2023–2024 from 313 insurance employees in Northern Cyprus using validated scales measuring proactive personality, innovative work behavior, task performance, and contextual performance. Participants completed self-report questionnaires, and the data were analyzed using SPSS 30.0 with correlation and regression analyses.

**Results:**

The results revealed that proactive personality was positively and significantly associated with innovative work behavior, task performance, and contextual performance. Innovative work behavior was also positively related to both dimensions of employee performance (task and contextual). These findings indicate that proactive dispositions and innovation-oriented behaviors co-occur with stronger performance outcomes in this service setting.

**Discussion:**

Overall, the findings underscore the managerial importance of fostering proactive dispositions to promote discretionary innovation and enhance both in-role and extra-role performance in insurance organizations. Given the cross-sectional and self-report nature of the data, the relationships should be interpreted as associative rather than causal. Future research should employ longitudinal designs and multi-source data to establish causality and generalizability.

## 1 Introduction

Rapid technological change, intensified competition, and increasing customer expectations have fundamentally reshaped contemporary work environments, particularly in service-oriented industries. In such contexts, organizations rely not only on formal job skills but also on employees’ psychological characteristics that enable initiative, adaptability, and discretionary contribution. From a managerial psychology perspective, understanding how stable individual differences translate into innovative behavior and performance outcomes has become a central concern for both scholars and practitioners.

Among these individual differences, proactive personality has emerged as a key construct in organizational psychology. Proactive personality reflects a dispositional tendency to take initiative, identify opportunities, and persist in efforts to effect meaningful change in one’s work environment (Bateman and Crant, 1993). Unlike reactive orientations, a proactive personality emphasizes self-initiative and future-oriented action, making it particularly relevant in dynamic, customer-facing service settings. Prior research has linked proactive personality to a range of desirable outcomes, including job performance, career success, and innovation-related behaviors (Crant et al., 2016).

A growing body of managerial psychology research suggests that proactive individuals are especially likely to engage in innovative work behavior, defined as the intentional generation, promotion, and implementation of novel ideas aimed at improving work processes, services, or outcomes (Janssen, 2000). Innovative work behavior is not limited to formal innovation roles; rather, it represents a form of discretionary behavior through which employees contribute psychologically and behaviorally beyond prescribed job requirements. Proactive employees, through their initiative and persistence, are more likely to identify opportunities for improvement and act on them, even in the absence of explicit managerial directives.

At the same time, employee effectiveness is increasingly conceptualized as a multidimensional construct, encompassing both task performance and contextual performance (Borman and Motowidlo, 1997). Task performance refers to the execution of core job duties. In contrast, contextual performance captures discretionary behaviors such as cooperation, responsibility, and voluntary effort that sustain the social and psychological work environment. Managerial psychology research emphasizes that personality traits may differentially relate to these performance domains, with proactive tendencies often showing stronger associations with contextual and discretionary aspects of performance (Cropanzano et al., 2001; Qandeel and Kuráth, 2025).

Despite extensive research on proactive personality, several gaps remain. First, much of the existing literature has focused on manufacturing, technology, or healthcare contexts (Fuller and Marler, 2009), with comparatively limited attention to financial and insurance services, where innovation is often incremental, relational, and constrained by regulation. Recent studies continue to emphasize digital, educational, and hospitality contexts (Lewaherilla et al., 2024; Zia et al., 2025; Barkat et al., 2024; Chen, 2023) rather than regulated financial industries, despite the sector’s economic significance (Ministry of Finance, Cyprus, 2024). Second, empirical studies examining proactive personality, innovative work behavior, and performance simultaneously remain relatively scarce, particularly in small, service-based economies. While meta-analytic evidence confirms links between proactive personality and task performance/organizational citizenship behavior (Zhang et al., 2022), the simultaneous examination of these constructs alongside innovative work behavior in service contexts is underdeveloped (Dong et al., 2024). Cross-cultural personality research further suggests that proactive tendencies may manifest differently across contexts (Luo et al., 2025), yet validation studies for performance measures remain concentrated in Western and Asian settings (Galanis et al., 2024; Yildiz and Şen, 2024; Nieva et al., 2025). Such contexts may activate proactive tendencies differently, as employees often operate within close-knit organizational structures and resource-limited environments that heighten the importance of initiative and discretionary contribution (Rienda et al., 2025; Demerouti, 2025).

The insurance sector provides a theoretically meaningful context for extending research on proactive personality in managerial psychology. In Cyprus, financial and insurance activities constitute approximately 8% of GDP growth (Ministry of Finance, Cyprus, 2024), yet the Northern Cyprus (TRNC) insurance market remains empirically underexamined (Dagli Insurance, 2023). High customer interaction, standardized procedures, and performance pressures related to service quality and trust characterize insurance work. Regulatory focus theory suggests that such constrained environments may activate prevention-focused motivational systems (Dong et al., 2024; Higgins, 2012), potentially altering how proactive personality translates into innovative behavior. Within this context, proactive personality may play a crucial role in shaping how employees adapt processes, engage clients, and contribute beyond formal role expectations. Examining these relationships in Northern Cyprus further allows exploration of proactive behavior in a relatively underexamined but theoretically relevant service environment.

Accordingly, this study examines associations among proactive personality, innovative work behavior, and two key performance domains—task and contextual performance—among insurance employees in Northern Cyprus. Drawing on Conservation of Resources and Job Demands-Resources theories (Demerouti, 2025), we investigate how dispositional proactivity translates into innovation and performance in a resource-constrained, regulated service setting. Adopting a managerial psychology perspective, the study clarifies how a stable dispositional characteristic relates to innovation-oriented behavior and performance outcomes in everyday work settings. In practice, the findings offer managers and human resource professionals insights into recruitment, development, and leadership practices that foster initiative, innovation, and discretionary performance in service organizations.

## 2 Theoretical background and hypothesis development

### 2.1 Proactive personality as a distal psychological resource

Proactive personality reflects a stable individual disposition characterized by self-initiated action, opportunity recognition, and persistent efforts to bring about meaningful change in one’s work environment (Bateman and Crant, 1993). Within managerial psychology, proactive personality is understood as a dispositional psychological resource that shapes how employees interpret, respond to, and actively influence their work contexts. Rather than reacting passively to situational demands, proactive individuals are inclined to anticipate future challenges, initiate improvements, and persist in goal-directed behavior.

Drawing on Proactive Motivation Theory (Xu et al., 2025), proactive personality can be conceptualized as a distal antecedent that energizes motivational states, including goal generation, proactive role orientation, and sustained effort. From this perspective, proactive individuals are more likely to translate dispositional initiative into concrete workplace behaviors when situational cues allow for discretion and self-directed action.

### 2.2 Proactive personality and innovative work behavior

Innovative work behavior refers to employees’ intentional generation, promotion, and implementation of novel ideas to improve work processes, services, or outcomes (Janssen, 2000). From a managerial psychology perspective, innovative work behavior represents a form of discretionary contribution through which employees actively shape their work environment. Notably, innovation in service sectors often manifests as incremental improvements, adaptive problem-solving, and relational adjustments rather than radical change.

Prior research suggests that proactive individuals are especially likely to engage in innovative work behavior because they actively seek opportunities for improvement, challenge existing practices, and persist despite obstacles (Crant et al., 2016). This pattern is consistent with earlier work by Wang et al. (2025), who demonstrated that proactive personality is closely linked to self-initiated, change-oriented behavior at work, particularly in contexts that offer opportunities for autonomy and problem-solving. From a trait activation perspective (Tett and Burnett, 2003), organizational contexts characterized by uncertainty, customer interaction, and performance pressure—such as the insurance sector—are likely to activate proactive tendencies, making innovation-oriented behavior a natural expression of proactive personality.

Accordingly, a proactive personality is expected to be positively associated with innovative work behavior.

Beyond the foundational studies mentioned earlier, recent empirical research further reinforces the link between proactive personality and innovative work behavior by elucidating the psychological mechanisms and theoretical underpinnings of this relationship. While early studies often faced limitations such as cross-sectional designs, single-sector samples, and dependence on self-reported data, the overall evidence steadily shows a strong, positive connection between proactive personality and innovation-driven actions across various organizational settings.

For example, Dai et al., 2024 provide recent empirical evidence that proactive personality influences innovative work behavior both directly and indirectly through work-related flow. Through quantitative methods and structural equation modeling, their research indicates that employees with proactive traits tend to experience greater absorption, intrinsic motivation, and cognitive engagement in their tasks, which subsequently increases their chances of creating, promoting, and executing new ideas. Significantly, this study goes beyond merely linking traits and behaviors by highlighting work-related flow as a mediating psychological factor, thereby supporting the idea that proactive personality serves as a distal psychological resource that fosters innovation through motivational and emotional mechanisms.

Similarly, Li et al. (2022) combine Schumpeter’s Theory of Innovation with the Broaden-and-Build Theory to offer a richer explanation of how proactive personality leads to innovative work behavior. Using empirical data and multivariate statistical methods, their results show that proactive individuals are more apt to spot opportunities, accept change, and activate positive psychological resources that broaden their cognitive and behavioral options. By merging economic innovation theory with positive psychology, this research presents a multi-layered conceptual framework in which proactive personality not only directly encourages innovative behavior but also enhances employees’ ability to develop lasting personal and social resources that support ongoing innovation.

When these two studies are evaluated together, it is evident that proactive personality supports innovative work behavior through motivational, cognitive, and emotional mechanisms; therefore, the relationship has a multidimensional structure rather than a one-dimensional one. Summarizing these findings, along with their methodological approaches and main results in the literature section, will clearly demonstrate that the relationship between proactive personality and innovative work behavior is grounded in solid theoretical and empirical foundations; it will also more closely align the conceptual model of the current study with the international literature.

While prior research has established positive associations between proactive personality, innovative work behavior, and performance outcomes, several important gaps remain. First, much of the existing literature has examined these constructs either in isolation or through partial models, often focusing on the link between proactive personality and innovation, or between innovation and performance, rather than testing all three constructs simultaneously within a unified framework. Recent integrative reviews note that multi-path dispositional–behavioral–performance models remain relatively scarce and call for more comprehensive empirical testing of proactive personality within full structural frameworks (Li and Wu, 2025; Aloulou et al., 2025). This limits our understanding of how proactive dispositions, innovation-oriented behaviors, and multidimensional performance outcomes coexist in everyday organizational settings.

Second, the majority of empirical studies have been conducted in large-scale economies or in manufacturing, technology, and healthcare sectors, where innovation is frequently formalized and structurally supported. Emerging 2025–2026 research emphasizes that service-based and regulation-intensive sectors remain comparatively underrepresented in innovation personality research, particularly in small and peripheral economies (Al Nahyan et al., 2025; Cucculelli et al., 2026). Comparatively little attention has been devoted to small, service-based, regulation-intensive markets where innovation is incremental, relational, and embedded in frontline service interactions.

Third, the contextual boundary conditions under which proactive personality is activated and translated into observable performance remain underexplored, particularly in small economies characterized by high relational interdependence and limited organizational slack. Recent trait-activation and contextualized personality studies argue that proactive dispositions are highly sensitive to situational cues, yet empirical work examining micro-contextual activation in compact economies is still emerging (Billett and Le, 2025; Swieai et al., 2025; Li et al., 2026). Addressing these gaps, the present study integrates proactive personality, innovative work behavior, and both task and contextual performance within a single empirical model and examines these relationships in the under-researched insurance sector of Northern Cyprus. By doing so, it contributes to managerial psychology literature by clarifying how dispositional initiative operates as a micro-foundation of innovation and performance in compact, service-driven, and reputation-sensitive market environments.

*Hypothesis 1*. A proactive personality is positively associated with innovative work behavior.

### 2.3 A proactive personality and employee performance

Employee performance is widely conceptualized as a multidimensional construct encompassing both task performance and contextual performance (Borman and Motowidlo, 1997). Task performance refers to the effective execution of core job duties. In contrast, contextual performance captures discretionary behaviors such as cooperation, responsibility, and voluntary effort that support the social and psychological work environment.

Managerial psychology research suggests that a proactive personality may be particularly relevant to performance domains that allow discretion and personal initiative. In support of this view, Parker et al. (2006) argue that proactive dispositions are most likely to influence performance outcomes when employees have latitude to shape their work activities and respond flexibly to situational demands. Proactive employees are likely to enhance task performance by anticipating problems, seeking feedback, and adapting their work strategies. At the same time, their self-starting orientation and sense of responsibility are likely to foster contextual performance behaviors, including helping colleagues and voluntarily contributing to organizational functioning.

Empirical evidence across service and knowledge-intensive sectors supports positive associations between proactive personality and both task and contextual performance (Kalia and Bhardwaj, 2019; Fu et al., 2024). In the insurance sector, where service quality and relational effectiveness are critical, proactive dispositions may be especially valuable in sustaining both formal performance and discretionary contribution.

Accordingly, the following hypotheses are proposed:

*Hypothesis 2*. A proactive personality is positively related to task performance.

*Hypothesis 3*. A proactive personality is positively associated with contextual performance.

### 2.4 Innovative work behavior and employee performance

Innovative work behavior is also closely linked to employee performance outcomes. Employees who actively engage in idea generation and implementation often contribute to improved work processes, service quality, and problem-solving effectiveness, which are directly relevant to task performance. In addition, innovation-oriented behaviors frequently involve collaboration, knowledge sharing, and voluntary effort, thereby aligning closely with contextual performance.

From a managerial psychology standpoint, innovative work behavior can be understood as a complementary form of discretionary contribution that co-occurs with adequate performance. Rather than representing a tested causal mechanism, innovation-oriented behavior reflects a pattern of active psychological engagement with one’s work role and is associated with stronger performance outcomes.

Based on this reasoning, innovative work behavior is expected to be positively associated with both dimensions of employee performance.

*Hypothesis 4*. Innovative work behavior is positively related to task performance.

*Hypothesis 5*. Innovative work behavior is positively related to contextual performance.

## 3 Study overview

While the theoretical arguments above outline the general links among proactive personality, innovative work behavior, and employee performance, their significance becomes clearer when applied to a specific service setting. The insurance industry in Northern Cyprus is a small, highly relational market with strict regulations, characterized by close customer interactions, minimal organizational layers, and resource limitations typical of small economies. In these settings, employees often take on multiple roles, manage personalized client portfolios, and handle service recovery directly. These structural traits emphasize the need for discretionary initiative, adaptive problem-solving, and strong relational skills. As a result, a proactive personality not only reflects a dispositional trait but also acts as a practical necessity for maintaining service quality, building client trust, and staying competitive in a market driven by reputation. Viewing the following hypotheses through this lens offers a deeper understanding of how proactive traits and innovation behaviors lead to tangible performance results in Northern Cyprus’s insurance industry.

Institutional reports suggest that Northern Cyprus’s insurance sector functions within a small yet competitive service economy marked by limited market size, high customer concentration, and a reliance on relational trust. The Insurance and Reinsurance Companies Association of Northern Cyprus (2018) highlights that this sector includes a few active insurance companies competing fiercely in motor, health, and property insurance. In small economies, competition tends to focus more on service quality, responsiveness, customer loyalty, and relational stability than on price alone. Additionally, macroeconomic data from the State Planning Organization (2023) indicate that the services sector accounts for the majority of national income, placing pressure on frontline employees to perform. Under these conditions, employees’ initiative, problem-solving skills, and client-focused innovation are essential micro-foundations of competitiveness. Consequently, traits such as proactive personality and innovative work behavior are not only desirable but critical capabilities for organizational sustainability in a market that is both compact and reputation-sensitive.

From a theoretical perspective, this contextual relevance is further clarified by Trait Activation Theory and the Resource-Based View (RBV). Trait Activation Theory suggests that personality traits become evident when situational cues indicate their importance (Tett and Burnett, 2003). In Northern Cyprus’s insurance sector—marked by uncertainty, frequent customer interactions, and adaptable role requirements—strong cues activate proactive traits. At the same time, the Resource-Based View views human capital factors such as proactive personality and innovation-driven behavior as valuable, rare, and difficult to copy, thereby helping maintain competitive advantage (Pontillo et al., 2025). When employees regularly solve problems creatively and contribute voluntarily, these micro-level psychological traits build up into organizational capabilities. Including proactive personality and innovative work behavior in the Northern Cyprus insurance context not only bolsters the empirical basis of the study but also links the hypotheses to well-established theories that connect individual traits to strategic results.

This study employed a relational survey design, a quantitative method commonly used to examine associations between personality traits and performance outcomes. The approach was considered appropriate as it enables the identification of associations among variables of interest.

## 4 Study 1: field experiment

### 4.1 Procedure and sample

Data were collected between October 5, 2023, and January 5, 2024. Before data collection, participants were informed about the study’s purpose, scope, and confidentiality, and their voluntary participation was obtained through written consent. Questionnaires were distributed in both paper and electronic formats to facilitate accessibility.

The study population consisted of employees working in insurance companies operating in Northern Cyprus in 2023. According to sectoral records obtained from the relevant regulatory authority and industry reports, the total number of employees working in licensed insurance companies during the data collection period was approximately 750–800, including administrative staff, technical personnel, and sales representatives. Thus, the study’s target population is finite and relatively small.

In determining the sample size, commonly accepted sampling guidelines for finite populations were applied. At a 95% confidence level and a 5% margin of error, the minimum required sample size for a population of approximately 800 employees is approximately 260 respondents. Furthermore, from a multivariate analysis perspective, recommendations suggesting a minimum of 10–15 participants per estimated parameter and at least 200 cases for structural analyses were also considered. In this context, the final sample of 313 participants exceeded the minimum thresholds suggested both by sampling theory and multivariate statistical guidelines, thereby enhancing the statistical power and generalizability of the findings within the defined population.

A total of 313 participants were selected through randomized sampling. The sample comprised 206 males (65.8%) and 107 females (34.2%) employees. The mean age of participants was 38.5 years (*SD* = 9.2). In terms of educational background, 45.7% held bachelor’s degrees, 28.1% had completed postgraduate studies (master’s or doctoral degrees), 21.4% were high school graduates, and 4.8% had other educational qualifications. Regarding organizational tenure, 35.1% had 1–4 years of experience, 28.4% had 5–8 years, 21.4% had 9–15 years, and 15.1% had 16 or more years. Job positions included staff employees (62.3%), lower-level managers (18.5%), middle-level managers (12.8%), and senior managers (6.4%). The sample thus included employees with diverse demographic backgrounds, job positions, and work experience, ensuring representation of different groups within the sector.

### 4.2 Measures

All constructs were measured using previously validated scales widely employed in organizational psychology research. Unless otherwise stated, items were rated on a five-point Likert-type scale ranging from 1 (strongly disagree) to 5 (strongly agree). Higher scores indicate higher levels of the respective construct.

#### 4.2.1 Sociodemographic information form

A brief form developed by the researchers was used to collect background information, including gender, age, education level, organizational tenure, and job position. These variables were used to describe the sample and to explore potential demographic differences in proactive and performance-related outcomes.

#### 4.2.2 Proactive personality

Proactive personality was measured using the Proactive Personality Scale originally developed by Bateman and Crant (1993) and adapted into Turkish in prior validation studies. The scale captures individuals’ dispositional tendency to identify opportunities, initiate change, and persist in goal-directed action. Sample items include “I am constantly on the lookout for new ways to improve my life” and “If I see something I do not like, I fix it.” The scale demonstrated strong internal consistency in this study (Cronbach’s α = 0.84). Confirmatory factor analysis supported its unidimensional structure, consistent with previous research.

#### 4.2.3 Innovative work behavior

Innovative work behavior was assessed using the nine-item scale developed by Janssen (2000), which conceptualizes innovation as a three-stage process involving idea generation, idea promotion, and idea implementation. A sample item is “I create new ideas for difficult issues at work.” In line with prior studies, items were averaged to create a composite innovation score. The scale showed satisfactory reliability in this study (Cronbach’s α = 0.78).

#### 4.2.4 Task performance

Task performance was measured using a scale assessing the effectiveness with which employees carry out their formal job responsibilities. Items reflect the completion of core duties, quality of work, and achievement of assigned objectives. Participants rated statements such as “I adequately complete assigned duties” and “I fulfill the responsibilities specified in my job description.” Internal consistency was acceptable (Cronbach’s α = 0.81).

#### 4.2.5 Contextual performance

Contextual performance was assessed using items derived from the framework proposed by Borman and Motowidlo (1997), covering interpersonal facilitation (e.g., helping coworkers) and job dedication/voluntariness (e.g., putting in extra effort beyond formal requirements). A sample item includes “I voluntarily help others who have heavy workloads.” The scale demonstrated good internal reliability (Cronbach’s α = 0.86).

Overall, reliability coefficients for all study variables exceeded the commonly accepted threshold of 0.70, indicating satisfactory internal consistency.

### 4.3 Analytical approach

All instruments used in this study had established validity and reliability in prior research, and reliability was reconfirmed in the present sample (Cronbach’s alpha values are reported above in the Measures section).

Data were analyzed using the Statistical Package for the Social Sciences (SPSS) version 30.0. Descriptive statistics were used to summarize participants’ characteristics. Independent samples *t*-tests and ANOVA were conducted to examine differences across demographic groups. Correlation analyses were performed to identify relationships among variables, and multiple regression analyses were used to investigate the associations among proactive personality, innovative behavior, task performance, and contextual performance. The significance level was set at *p* < 0.05.

### 4.4 Ethical considerations

Ethical approval for the study was obtained from the Cyprus University of Health and Social Sciences Research Ethics Committee (Approval No: KSTU/2023/214). Participants were assured of confidentiality and anonymity, and all procedures complied with ethical research standards.

## 5 Results

**Figure 1:**
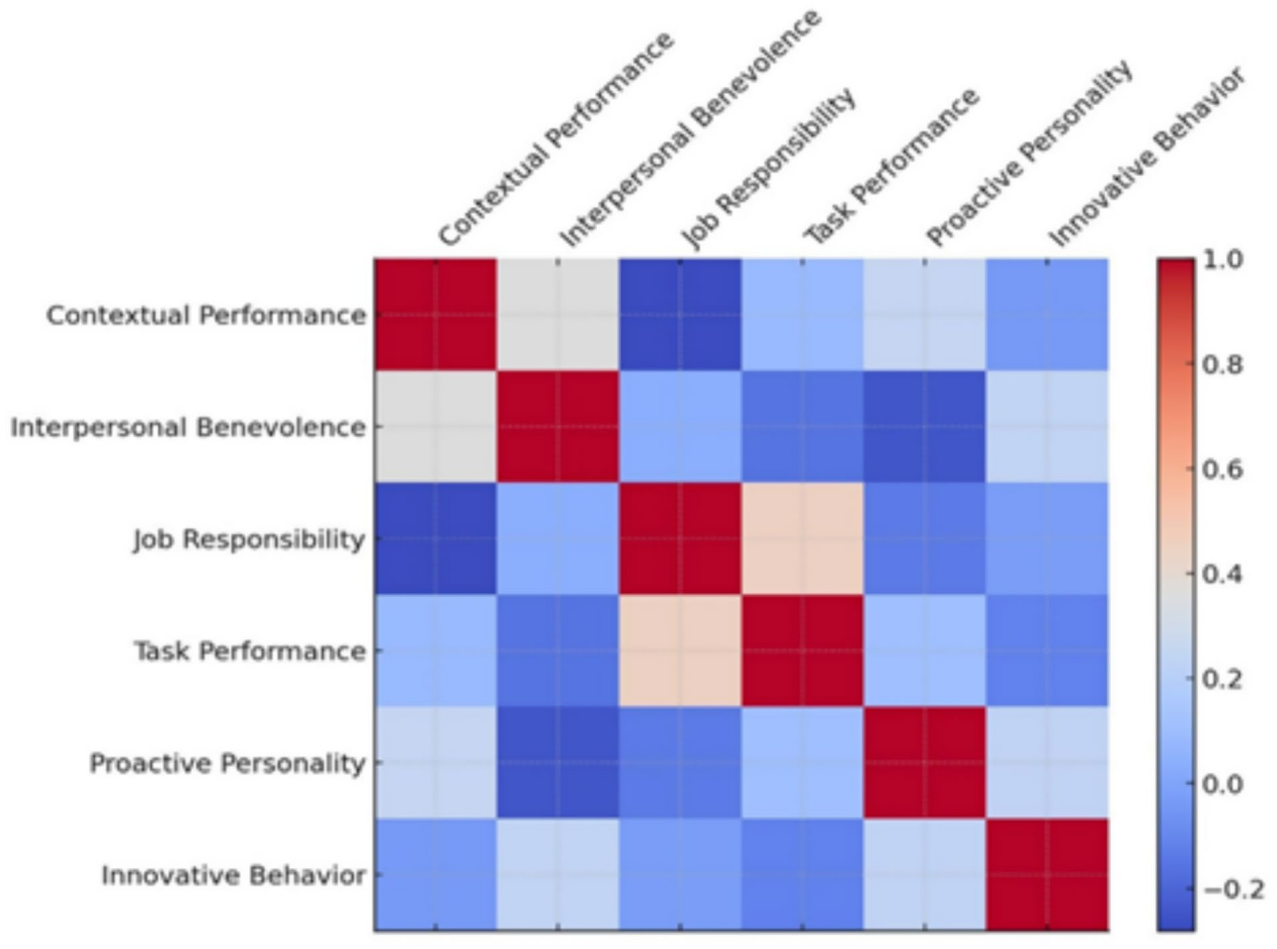
Correlation heatmap of study variables.

Descriptive statistics indicated that participants reported relatively high levels of proactive personality, innovative work behavior, task performance, and contextual performance. Mean scores suggested that discretionary and responsibility-related aspects of performance were particularly salient in the sample. Table 1 and Figure 1 present the means and standard deviations for all study variables.

**Table 1:** Descriptive statistics for study variables.

Variable	*N*	Mean	*SD*
Contextual performance	313	66.42	12.44
Interpersonal benevolence	313	33.02	6.32
Job responsibility and voluntariness	313	33.4	6.53
Task performance	313	16.81	2.86
Proactive personality	313	52.53	7.58
Innovative behavior	313	23.96	4.35

Independent-samples *t*-tests indicated no statistically significant gender differences in proactive personality, innovative work behavior, task performance, or contextual performance (*p* > 0.05) (Table 2). Similarly, marital status did not significantly differentiate innovative work behavior or task performance. However, divorced or separated employees reported higher levels of proactive personality, interpersonal benevolence, and job responsibility/voluntariness compared to single and married employees (*p* < 0.05).

**Table 2:** Group differences by gender (independent samples *t*-test).

Variable	Female (*N* = 107) Mean (*SD*)	Male (*N* = 206) Mean (*SD*)	*t*	*p*
Contextual performance	65.45 (13.54)	66.92 (11.83)	−0.95	0.343
Interpersonal benevolence	32.80 (6.76)	33.13 (6.10)	−0.41	0.679
Job responsibility and voluntariness	32.64 (7.12)	33.79 (6.19)	−1.41	0.16
Task performance	17.09 (2.52)	16.67 (3.02)	1.33	0.184
Proactive personality	51.93 (7.72)	52.84 (7.51)	−0.99	0.319
Innovative behavior	23.52 (4.46)	24.19 (4.28)	−1.28	0.203

One-way ANOVA results showed that tenure and organizational position were associated with selected outcome variables. Employees with 1–4 years of experience reported higher task and contextual performance than those with longer tenure, while those with 5–8 years of experience reported higher innovative work behavior. Proactive personality scores were highest among employees with nine or more years of experience. In terms of organizational position, lower-level managers reported significantly higher contextual performance and interpersonal benevolence than staff, middle-level managers, and senior managers. Educational background was also significant, with postgraduate employees reporting stronger proactive personality, innovative work behavior, and task performance than those with lower educational attainment.

Pearson correlation analyses revealed several significant relationships among the study variables (see Table 3). Proactive personality was positively correlated with contextual performance and innovative work behavior, indicating that employees with stronger proactive dispositions also reported higher levels of discretionary performance and innovation-oriented behavior. Task performance showed a strong positive correlation with job responsibility and voluntariness, while its correlation with proactive personality was modest.

**Table 3:** Correlation matrix of study variables.

Variable	Contextual performance	Interpersonal benevolence	Job responsibility and voluntariness	Task performance	Proactive personality	Innovative behavior
Contextual performance	1.0	0.359	0.281	0.089	0.251	−0.037
Interpersonal benevolence	0.359	1.0	0.037	−0.16	−0.248	0.241
Job responsibility and voluntariness	0.281	0.037	1.0	0.449	−0.136	−0.026
Task performance	0.089	−0.16	0.449	1.0	0.11	−0.12
Proactive personality	0.251	−0.248	−0.136	0.11	1.0	0.233
Innovative behavior	−0.037	0.241	−0.026	−0.12	0.233	1.0

Notably, innovative work behavior was positively associated with interpersonal benevolence, suggesting that innovation-oriented employees may also be more inclined toward cooperative and supportive behaviors at work.

**Figure 2:**
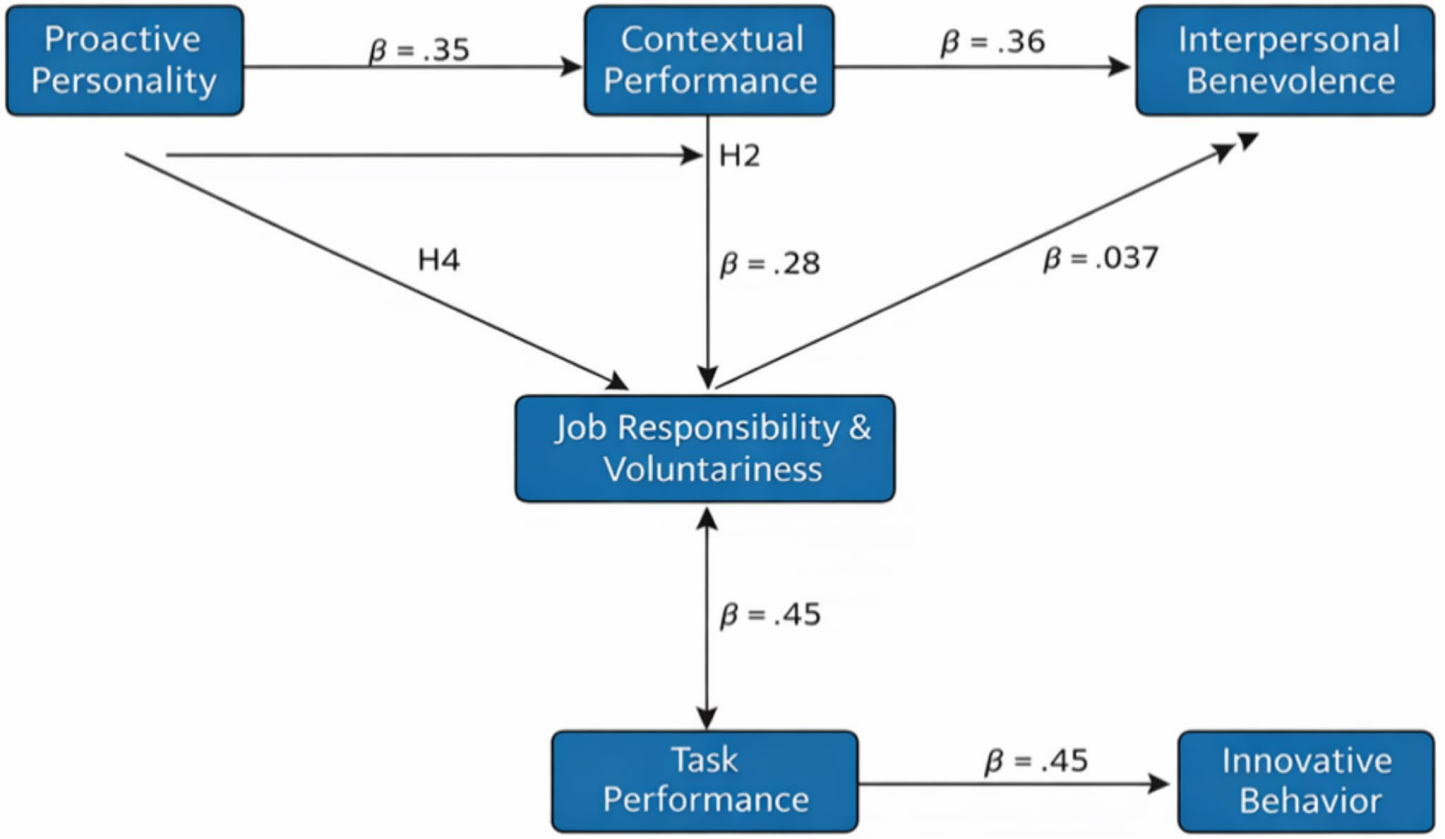
Path diagram of regression results.

Multiple regression analyses were conducted to examine whether proactive personality significantly predicted innovative work behavior, task performance, and contextual performance. Proactive personality emerged as a significant positive predictor of innovative work behavior (β = 0.36, *p* < 0.001), accounting for 17% of the variance in this behavior. In addition, the proactive personality significantly predicted task performance (β = 0.40, *p* < 0.001) and contextual performance (β = 0.42, *p* < 0.001), accounting for 19 and 23% of the variance, respectively (Figure 2).

Although the bivariate correlation between proactive personality and task performance was relatively modest, proactive personality demonstrated a stronger predictive relationship in the multivariate regression model. This pattern suggests that proactive personality explains unique variance in task performance when examined alongside other variables, a phenomenon observed in organizational research, where personality traits exert their influence more clearly in multivariate analytical contexts than in simple bivariate associations.

Innovative work behavior was also positively associated with both task and contextual performance, indicating that employees who reported higher engagement in innovation-oriented behaviors also tended to report stronger performance outcomes.

Overall, the results indicate that proactive personality is consistently associated with innovative work behavior and both task and contextual performance among insurance employees. The findings further suggest that innovation-oriented behavior co-occurs with stronger performance outcomes, highlighting the close interrelationship between personality, discretionary behavior, and performance in service-oriented work contexts.

## 6 Discussion

The observed associations between proactive personality and innovative work behavior are consistent with prior research in organizational and managerial psychology, which has repeatedly shown that individuals with proactive dispositions are more likely to engage in initiative-taking and improvement-oriented behaviors (Crant et al., 2016; Tuna and Işık Demirarslan, 2023). The present findings extend this line of research by demonstrating that such associations also hold in the insurance sector, where innovation is typically incremental and embedded within service delivery and relational processes rather than radical transformation. This suggests that proactive personality may be particularly relevant for sustaining continuous improvement and adaptive problem-solving in regulated service environments.

Similarly, the positive relationships between proactive personality and both task and contextual performance align with evidence from other service-oriented contexts (Kalia and Bhardwaj, 2019; Fu et al., 2024). Notably, the association with contextual performance appeared more pronounced, indicating that proactive tendencies may be more readily expressed through discretionary and relational behaviors. This pattern refines existing understanding by suggesting that the performance benefits of proactive personality are especially salient in domains that allow behavioral discretion, such as cooperation, responsibility, and voluntary contribution, which are central to service quality and customer experience in insurance work.

At the same time, the findings offer insight into inconsistencies reported in previous studies that have identified weaker or non-significant relationships between proactive personality and performance outcomes (Alanoğlu and Karabatak, 2018; Demir and Arabacı, 2021). Such discrepancies may reflect differences in occupational roles, organizational norms, or performance measurement approaches that shape the extent to which proactive dispositions are activated and translated into observable behavior. In the present context, frequent customer interaction and accountability for service outcomes may heighten opportunities for proactive behavior, thereby strengthening its association with both innovation-oriented and performance-related outcomes.

The demographic patterns observed in this study further support the view that proactive personality operates within specific contextual and developmental conditions. In line with prior research, tenure and educational background appeared to shape how proactive tendencies were expressed, with higher levels of education and experience associated with stronger proactive and innovation-oriented behaviors (Woods et al., 2018). Taken together, these findings suggest that proactive personality does not function uniformly across settings, but interacts with contextual features and individual development to shape discretionary behavior and performance in service organizations.

The observed associations between proactive personality and innovative work behavior are consistent with previous findings in the organizational and managerial psychology literature, which have repeatedly shown that individuals with proactive tendencies are more likely to exhibit initiative-taking and improvement-oriented behaviors (Crant et al., 2016; Tuna and Işık Demirarslan, 2023). The current findings extend this line of research by revealing that this relationship is also valid in the insurance sector, which is often characterized by incremental innovations embedded in service delivery and relational processes rather than radical transformations. This suggests that proactive personality can be a critical psychological resource in maintaining continuous improvement and adaptive problem-solving, especially in regulated service settings.

However, the current study not only validates previous findings but also addresses an important gap in the literature by integrating proactive personality, innovative work behavior, and multidimensional performance indicators within a single model. Previous research has mostly examined these variables at the bilateral level but has paid limited attention to their interactions within a holistic framework, especially in small-scale service economies (Dai et al., 2024). In this respect, the study makes a theoretical contribution by combining Trait Activation Theory (Tett and Burnett, 2003), which explains how personality traits are transformed into behavior through contextual cues, with the relational nature of performance in the service sector.

Similarly, positive associations between proactive personality and both task and contextual performance are consistent with findings in other service-oriented contexts (Kalia and Bhardwaj, 2019; Fu et al., 2024). In particular, the relationship with contextual performance is more pronounced, indicating that proactive tendencies are expressed through behaviors that require more discretion and relational interaction. This pattern deepens the current understanding, revealing that the effects of proactive personality on performance are particularly pronounced in areas involving behavioral freedom, such as collaboration, responsibility, and voluntary contribution. This finding is particularly important in the sectoral context, as service quality and customer experience in insurance largely depend on employees’ voluntary efforts.

In addition, these results contribute to the ongoing debate about which performance dimension is stronger in the personality-performance relationship. Meta-analytic studies suggest that personality traits show stronger associations, particularly with off-role and voluntary behaviors (Busseri and Erb, 2024). Current findings support this view, revealing that the impact of proactive personality on contextual performance is more pronounced compared to task performance. Thus, the study develops a more nuanced interpretation that considers the multidimensional nature of performance.

On the other hand, the findings also shed light on the inconsistencies related to weak or meaningless relationships reported in some previous studies (Alanoğlu and Karabatak, 2018; Demir and Arabacı, 2021). Such differences may arise from variability in professional roles, organizational norms, or performance measurement approaches. In the current context, intense interaction with the customer and high level of accountability for service outputs may have increased the likelihood of proactive behaviors and paved the way for these trends to be more strongly associated with both innovative behavior and performance indicators.

This suggests that proactive personality is not a deterministic factor in itself, but rather takes on meaning in interaction with contextual opportunity structures and organizational demands. In service economies where small, relational networks are dominant, the visibility and impact of individual initiative may be higher, allowing proactive traits to be more directly reflected in behavioral outcomes. Therefore, the study offers an innovative perspective on the literature by revealing that proactive personality should be considered a context-sensitive psychological resource.

The demographic patterns observed in the study also support that proactive personality operates under certain contextual and developmental conditions. Consistent with previous research, proactive and innovation-oriented behaviors have been shown to strengthen as seniority and education level increase (Woods et al., 2018). This shows that proactive tendencies become more effective behavior when supported by cognitive resources, professional knowledge, and experience.

In conclusion, this study not only validates existing relationships but also fills an important gap in the literature by holistically testing the links among proactive personality, innovative work behavior, and multidimensional performance in a regulated, highly relational service sector. Thus, it clarifies the theoretical-level contextual boundary conditions and emphasizes the strategic importance of proactive features in applied managerial psychology.

## 7 Practical implications

From a managerial perspective, the findings underscore the value of recognizing proactive personality as a psychological resource in service organizations. Managers and human resources professionals may benefit from incorporating assessments of proactive tendencies into recruitment and selection processes, particularly for roles that require adaptability, client interaction, and discretionary effort. Behavioral interviews and situational judgment tests that assess initiative-taking and problem-solving orientation may be beneficial.

In addition, organizations can support proactive and innovative behaviors by creating work environments that encourage idea sharing, autonomy, and psychological safety. Training and development initiatives that emphasize self-leadership, continuous improvement, and creative problem-solving may further enhance the expression of proactive dispositions. Significantly, managerial support and recognition of discretionary contributions can reinforce employees’ willingness to engage in contextual and innovation-oriented behaviors that benefit both individual performance and organizational effectiveness.

In addition, future studies may contribute to refining and measuring employee performance by adopting multidimensional performance frameworks. For instance, Nieva et al., (2025) conceptualize employee performance as comprising task, contextual, and adaptive performance, and empirically validate this three-dimensional structure. While the present study focused on task and contextual performance, incorporating adaptive performance—defined as employees’ ability to adjust to changing work demands, technologies, and customer expectations—may provide a more comprehensive understanding of performance dynamics, particularly in rapidly evolving service sectors such as insurance. Expanding performance measurement in this direction would not only strengthen construct validity but also allow researchers to examine whether proactive personality and innovative work behavior differentially predict distinct performance dimensions. Such an approach could advance theoretical development in performance research and offer more fine-grained insights into how dispositional traits translate into diverse forms of effectiveness at work.

## 8 Conclusion

This study contributes to managerial psychology by clarifying how a proactive personality is associated with innovative work behavior and employee performance in a service-oriented, regulated context. The findings suggest that proactive dispositions are closely linked not only to innovation-oriented behaviors but also to both task-related and discretionary aspects of performance, highlighting the psychological importance of initiative and self-starting orientation in everyday work settings.

By examining these relationships in the insurance sector, the study extends existing research beyond commonly examined industries. It demonstrates that proactive personality remains relevant even in environments characterized by standardized procedures and regulatory constraints. Rather than diminishing the role of individual agency, such contexts may heighten the value of discretionary and innovation-oriented behaviors through which proactive employees contribute to organizational functioning.

From a managerial standpoint, the findings reinforce the importance of aligning recruitment, development, and leadership practices with psychological characteristics that support initiative and adaptability. Recognizing and nurturing proactive tendencies may help organizations enhance both performance and innovation in service roles where employee behavior plays a central role in customer experience and organizational effectiveness.

Overall, the study underscores the value of viewing proactive personality not merely as an individual trait but as a psychological resource that supports innovation and performance within complex organizational environments.

## Data Availability

The data that support the findings of this study are available from the corresponding author upon reasonable request.
